# Supplementary material for: Predictive design of crystallographic chiral separation
Source: Nat Commun. 2025 Aug 26;16:7977. doi: 10.1038/s41467-025-62825-4 (PMC12381160; doi:10.1038/s41467-025-62825-4)
Supplement: Supplementary file 1 — Supplementary Information [file 41467_2025_62825_MOESM1_ESM.pdf]

Supplementary Information: Predictive design of crystallographic chiral separation

S1 Molecular dynamics simulations of the acid-base pair

S1.1 Comment on initialising the acid-base pair as neutral species

The racemate-resolving agent pairs form a salt in the solid phase, i.e. the solid is composed of ions. As such, we initially played with the idea of initialising the simulations using the ion pair rather than the neutral molecules. However, running the simulation with the acid and base as charged species significantly reduces the explored conformations due to the strong electrostatic attractions. Furthermore, in our initial experiments with using the semi-empirical quantum mechanics PM7 method the two molecules would spontaneously transfer protons to neutralise. As such, all simulations were run using neutral molecules.

S2 Full list of solvent descriptors

Table S1 gives the full list of descriptors used in model. All descriptors were calculated using COSMO*therm* [1].

Descriptor	Units
Free energy of solvation	kcal mol^{-1}
$\log P_{\mathrm{octanol-water}}$	
Vapour pressure	bar
Henry's law constant of H_2 in solvent	bar
Liquid viscosity	centiPoise
σ_2-σ_6 (moments of the charge profile)	
1st-5th moments of hydrogen bond donor charge profile	
1st-5th moments of hydrogen bond acceptor charge profile	
Dielectric energy	kcal mol^{-1}
Energy gain from averaging surface charges	kcal mol^{-1}
Molecular van der Waals energy	kcal mol^{-1}
Ring correction energy	kcal mol^{-1}
Chemical potential of the solvent in the gas phase	kcal mol^{-1}
Total dipole moment	Debye

Table S1: The descriptors used for representing the solvent. All values were calculated using COSMO*therm* [1].

S3 Further model details

S3.1 Compression of the atom-density representation

Using our atom-density scheme, each atom is represented by a ≈ 7000 dimensional vector. Such high dimensionality slows down the training process and requires high memory allocation. As such, we compress these representations using a simple auto-encoder network [2] down to 20 dimensions as a preprocessing step. In our experiments, the compression task was well behaved and we achieved low errors with a variety of architectures. Here we used a two-layer network with 6000 hidden dimensions and LeakyReLU activation.

The auto-encoder was trained on a larger set of atomic environments, including environments from proprietary molecules. As such, the training data for the auto-encoder will not be made available, however, we include the model weights and another encoder trained just on the representations of the acid-base pairs.

S3.2 Model architecture

Figure S1 shows the full model architecture. A core design feature of the architecture is the built-in invariance with respect to the order of diastereomeric pairs. This is achieved by applying the transformer blocks to the mean and absolute difference of the acid-base pair representations.

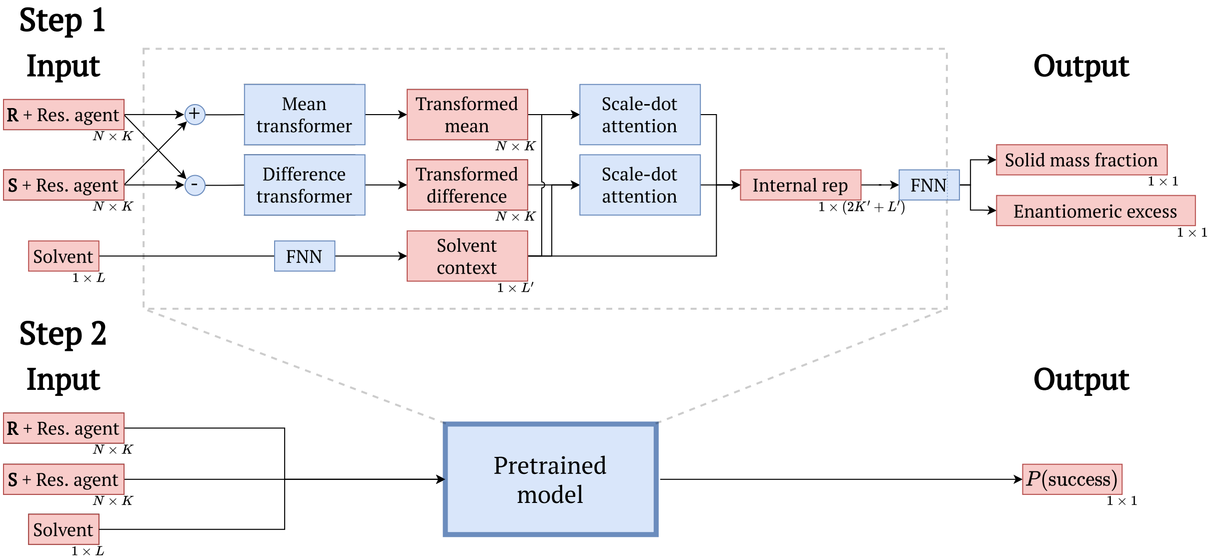

Figure S1: The model transforms the individual representations of the two enantiomers into two new tensors: One tensor contains the absolute difference between the two representations, and the other the mean of the representations. This allows the model to independently focus on the interaction differences and the overall structure of the acid-base pair. The scaled-dot attention layers use the solvent context vector as the query to generate solvent-aware representations. FNN = Feedforward Neural Network. The red colour indicates a tensor; the blue colour indicates a transformation. Subscripts indicate the shape of each tensor, batching dimension is omitted for clarity (N corresponds to the number of atoms, K the embedding dimension, L is the number of solvent descriptors, the primes indicate that some embedding dimensions might change while passing through an FNN).

S3.3 Training procedure

For the retrospective tests (Figure 4 and Section 5.1 in the main text), 5 models were trained according to the scheme in Figure S2. To ensure a fair evaluation, we grouped unique racemates into five sets, aiming for a roughly equal distribution of both the total number of successful resolutions and the total number of tested conditions across these groups. This stratification mitigates the bias that could arise from the uneven representation of racemates with varying levels of experimental investigation within our dataset (see Section S4.2), while still maintaining a scaffold-split design.

The two-stage training first involved training on 4 of the splits, followed by additional training only on the low-noise portions of those 4 splits. The models were then evaluated on the low-noise portions of the left-out splits. The 5 models arising from this training process were then used as an ensemble for the prospective predictions.

As noted in the main text, only 3% of the training data correspond to successful resolutions. To facilitate training, we used weighted sampling to construct batches during training. For classification, each batch of data was constructed to contain an equal number of successful and unsuccessful resolutions. For regression, each batch of data contains an equal number of samples from three partitions: $X < 33\%$, $33\% < X < 66\%$, and $X > 66\%$, where X is either mass fraction of the solid or the enantiomeric excess. In our experiments, weighted sampling significantly improved the rate of convergence during training and the overall performance of the model.

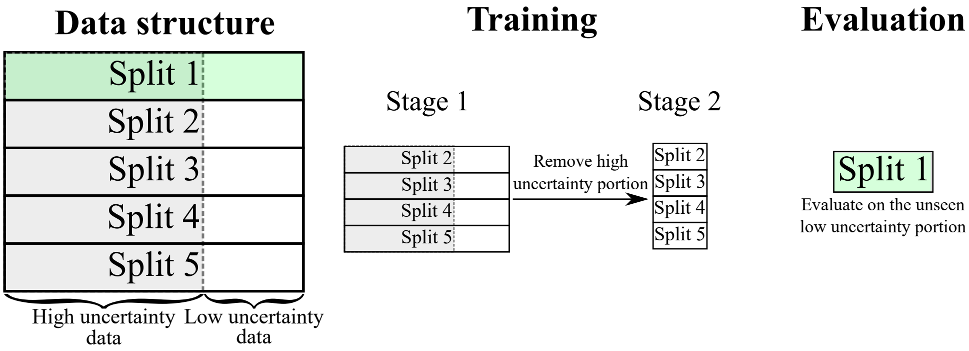

Figure S2: Data splits for the two-stage training. The figure illustrates on which data one of the five models was trained.

The models were trained using a single NVIDIA V100 with 32GB RAM, and the hyperparameters specified in Table S2. Note that the models used for prospective predictions, which used the trajectories generated with PM7 as detailed in Section 4.2, used a different learning rate schedule. Specifically, models used for prospective predictions used the learning rate given by:

$$\text{learning\_rate} \cdot \min(\text{epoch\_no}^{-0.5}, \text{epoch\_no} \cdot \text{warmup\_steps}^{-1.5})$$

with `warmup_steps=60`, a schedule taken from [3]. Further, models used for prospective predictions used 50,000 epochs for pretraining and 10,000 epochs for finetuning with no early stopping.

In both cases, the models were fully converged by the end of training.

Hyperparameter	Value	Comment
Transformer embedding dim	20	
Transformer layers	3	
Transformer no. heads	4	
Solvent context FNN no. layers	4	Residual network
Solvent context FNN input dim	26	Descriptors listed in Table S1
Solvent context FNN hidden dim	24	
Solvent context FNN out dim	20	
Cross-attention embedding dim	$104 \cdot 19$	Output of Solvent context FNN act as queries, output of the Transformer blocks as keys and values
Cross-attention no. heads	19	
Final FNN no. layers	2	
Final FNN input dim	$104 \cdot 19$	Takes in output of the cross-attention
Final FNN hidden dim	12	
Final FNN out dim	1 or 2	1 during fine-tuning, 2 during pretraining
Activation	LeakyReLU	Negative slope = 0.2
Batch size	128	
Dropout	0.32	
Learning rate	1e-4	
Learning rate schedule	OneCycle [4]	Max LR as shown above, other settings left to default
Optimiser	AdamW	Default PyTorch settings
Training epochs	1,000 with early stopping	Early stopping criteria – no validation set improvement for 100 epochs.

Table S2: Main model hyperparameters. Cross-attention refers to the scale-dot attention between the Transformed mean/difference and Solvent context in Figure 3 in the main text.

S3.4 Hyperparameter tuning

Hyperparameters were optimised using a Tree-structured Parzen Estimator strategy as implemented in Optuna [5]. For each trial, we sampled a configuration from a predefined search space encompassing learning rate, batch size, network architecture parameters (depth, hidden dimensions, attention heads), dropout probability, and activation function. Each configuration was evaluated by training a model and measuring binary cross-entropy loss on a dedicated validation set. Optuna's pruning mechanism was employed to accelerate the search by terminating unpromising trials early. The configuration yielding the lowest validation loss was subsequently used for our models presented in this work.

The optimization process identified a notably high number of cross-attention heads (19). While this could indicate the model's need to capture diverse interaction patterns between the acid-base pair and the solvent, it is more likely a consequence of the optimization balancing data fitting against the significant regularization from dropout ($p = 0.32$).

S3.5 Learning curve

To construct the learning curve, we use the average precision metric calculated using the *scikit-learn* Python package [6]. For classification problems with severe class imbalance, standard metrics, such as accuracy or area under the receiver-operator curve (AUC-ROC), can be misleading [7, 8]. These metrics can yield high scores for models that simply predict the majority class for all inputs. In contrast, the average precision emphasises the model's performance on the (minority) positive class and is equivalent to the area under the precision-recall curve.

The scaling behaviour of neural networks is best observed on a log-linear scale [9]. To generate the learning curve, we first reserved a 20 % portion of the data as a test set. We then trained models on four increasing fractions of the remaining data, equidistant on the log scale. For each fraction, we trained an ensemble of 10 models, where the training data were generated by random sampling with replacement. Throughout the experiment, we ensured that all subsets of the data contained the same fraction of positives as the overall data set.

S4 Extended training data analysis

S4.1 Data diversity

The compounds in our training data originate from historical pharmaceutical projects. Consequently, they are designed to be drug-like molecules, typically exhibiting low to medium molecular weight and generally adhering to Lipinski's Rule of Five [10]. Furthermore, the nature of diastereomeric resolution necessitates the presence of an acidic or basic functional group in these molecules.

To assess the diversity of this dataset within the broader context of drug-like chemical space, we compared our compounds to the GEOM-Drug dataset [11], a curated collection of drug-like molecules. Figures S3A and B show that our dataset exhibits similarity and drug-likeness distributions comparable to those observed in the GEOM-Drug dataset.

As a further validation, we used Morgan fingerprints to perform Principal Component Analysis (PCA) of both the GEOM-Drug and ChEMBL databases [12], with ChEMBL containing over 2 million drug-like compounds. Figure S4 shows that the two reference datasets have similar distributions in PCA space, with ChEMBL naturally having a larger extent due to its size. Our data, overlaid as points on these distributions, provide coverage of the high-density regions of both reference datasets.

These analyses show that the $\approx$ 450 unique chiral molecules within our data are representative of drug-like chemical space and provide adequate diversity for machine learning applications.

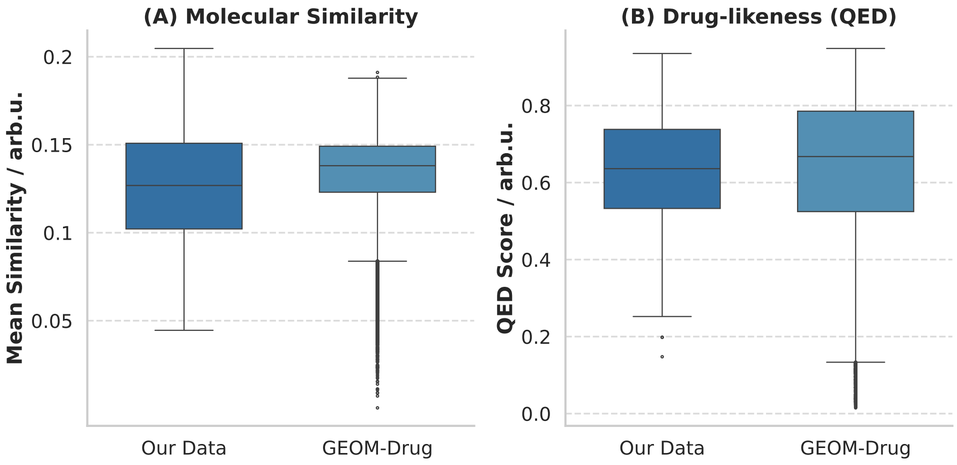

Figure S3: Diversity of training data compared to GEOM-Drug dataset. **A.** Comparison of mean similarity within the datasets as measured by Tanimoto similarity on Morgan fingerprints. **B.** Distribution of QED scores [13]. $n = 488$ for our data, and $n \approx 3 \cdot 10^5$ for GEOM-Drug data. The box plots show the median and the interquartile range (25th and 75th percentiles); the whiskers extend to extremes of the distribution (up to 1.5 the interquartile range), otherwise outliers are shown individually.

S4.2 Distribution of successful conditions

One reason for the high fraction of unsuccessful resolutions is the experimental screening process itself. Typically, high-throughput screening is conducted until a single successful resolution condition ("hit") is identified for a given racemate. Once a hit is found, the screening for that racemate often concludes. Further optimization of this hit, such as adjusting the racemate-to-resolving agent ratio, is a separate process and data from these targeted optimization screens are not included in our training dataset.

This screening strategy is directly reflected in Figure S5. As illustrated, the majority of racemates in our dataset have fewer than five successful resolutions identified. Furthermore, the total number

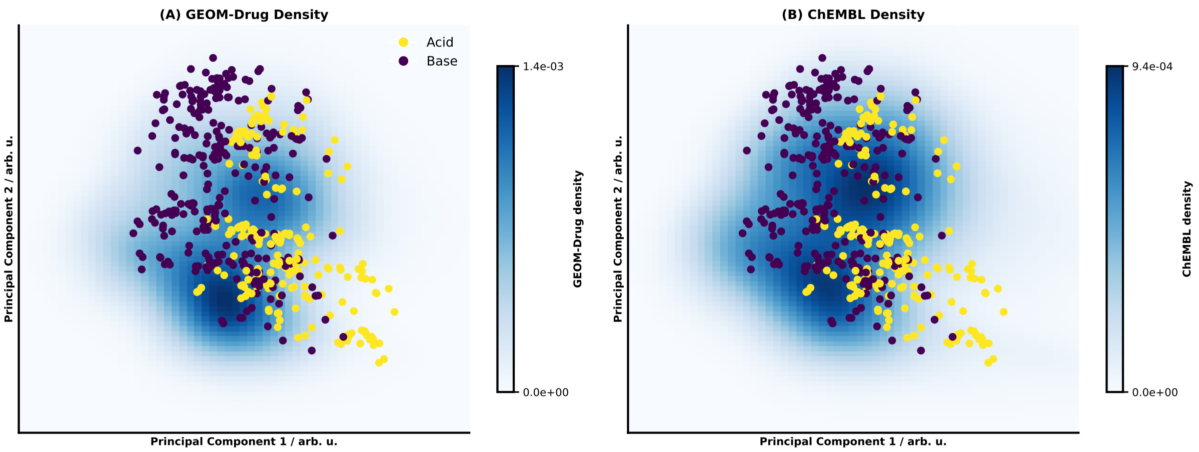

Figure S4: Principal Component Analysis (PCA) of reference datasets using Morgan fingerprints. **A.** PCA projection of GEOM-Drug compounds. **B.** PCA projection of ChEMBL compounds. Coloured points represent our training data projected onto the same PCA space. Both reference datasets show similar distributions, with ChEMBL exhibiting greater extent due to its larger size.

of conditions tested per racemate also varies significantly. The variability arises in part due to the inherent "resolvability" of each racemate but external factors, such as the importance of the racemate to a pharmaceutical project, also contribute.

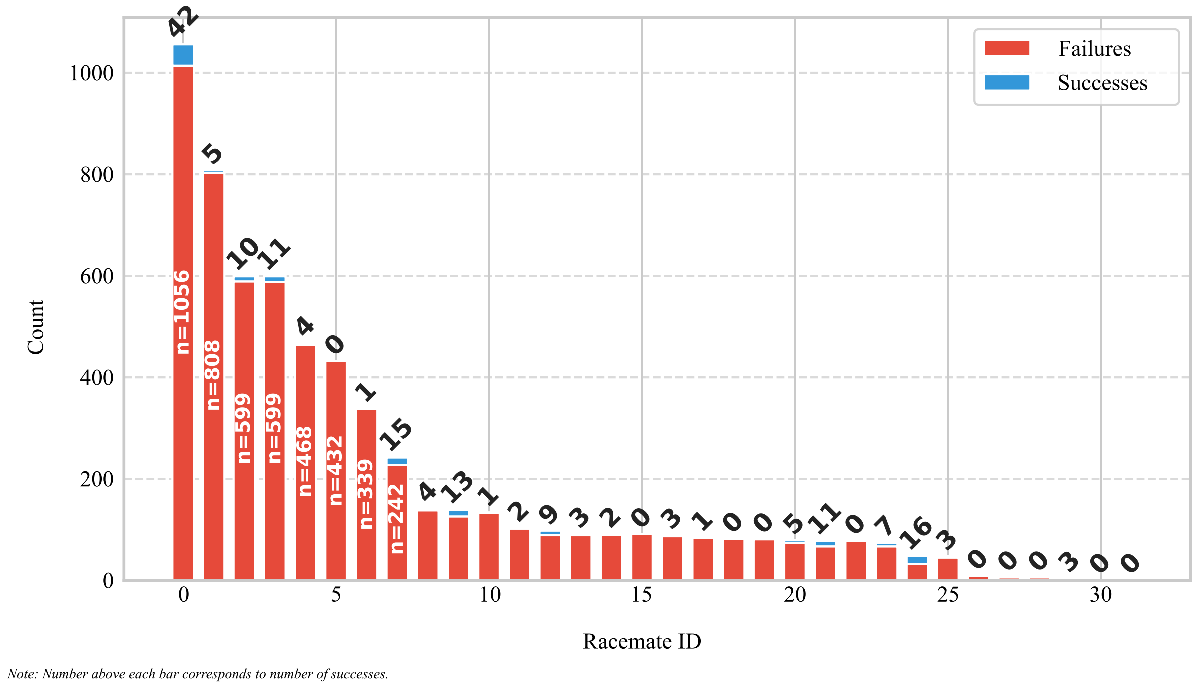

Figure S5: Distribution of successful resolution conditions per racemate.

S4.3 Distribution of figure of merit

To better visualise the overall outcomes of the resolution experiments, we consider the distribution of the figure of merit $z =$ solid yield $\cdot$ enantiomeric excess. Figure S6 shows that the vast majority of resolutions had a $z < 0.05$. This cliff in the distribution is also the cutoff chosen to classify resolutions as "hits" for training purposes, note that the fraction of resolutions which are practically useful is smaller still.

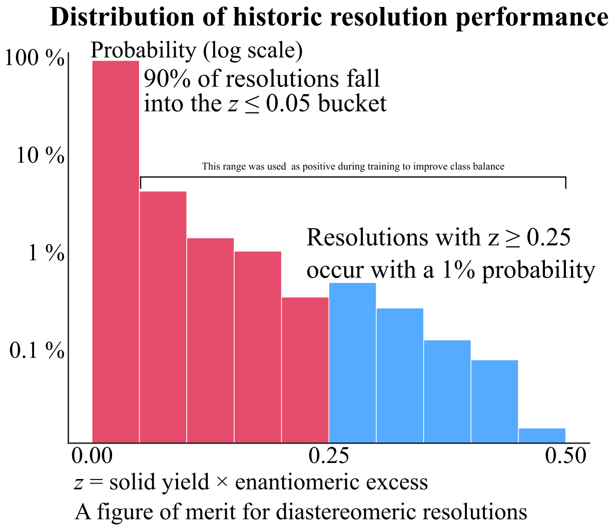

Figure S6: Distribution of data in terms of figure of merit.

S5 Retrospective scaffold split experiment details

S5.1 Modified 3DMolCSP model

As a more challenging baseline, we compared our model to 3DMolCSP [14], a deep neural network approach that, like our method, leverages 3D molecular conformations for representation learning. Originally, 3DMolCSP was developed for predicting retentions in chiral chromatography and thus requires a different set of inputs compared to our model.

To ensure a fair comparison, we adapted the original 3DMolCSP model to accept conformers of both enantiomers of the racemate and the separating agent as input. To maintain a similar parameter count to the original architecture, we utilized a shared encoder for all three molecules. Solvent information was added using one-hot encodings; for solvent mixtures, these encodings were summed, weighted by their respective volume fractions. This solvent encoding was then appended to the output of the 3DMolCSP encoder before being passed to the decoder.

The modified 3DMolCSP models were trained using the same train-test splits as our approach but using the original 3DMolCSP training script. In addition, we also performed two-step training just as for our approach. The results shown in Figure 4 correspond to a two-step training where a regression model was finetuned as regression again, which is the version that had the best overall metrics as shown in Table S3 below.

S5.2 Morgan Encoding details

Morgan fingerprints were generated with RDKit [15] using a chirality flag set to True, a radius of 2 (equivalent to ECFP4), and 1024 bits.

S5.3 Extended model comparison

Beyond the enrichment curves presented in Section 2.3 of the main text, we further evaluated the different approaches using standard classification metrics. Metrics such as F1-score, precision, and recall are sensitive to the probability threshold chosen for the positive class. While enrichment plots for regression-trained models were generated using a figure of merit ($z = \text{m.frac} \cdot \text{e.e.}$) to rank resolutions, this metric lacks a definitive boundary between positive and negative classes. Therefore, to enable a direct comparison across all models, we established a probability threshold by selecting the highest value that classified 100 conditions as positive. This serves as a suitable proxy, given our focus on early recognition performance.

Table S3 compares the performance of multiple baseline models, our approach and our implementation of 3DMolCSP. Overall, our approach, employing a two-step training strategy of initial regression training followed by classifier fine-tuning, achieves superior overall performance. While 3DMolCSP models exhibit precision and recall for the positive class comparable to the Random Forest baselines, their

AUC ROC values are similar to - and on one occasion exceed - our best models. This suggests that 3DMolCSP models, while less effective in early recognition, are proficient at identifying conditions with a low likelihood of success.

We hypothesize that the observed performance difference stems from representational disparities. The two neural network architectures likely have similar expressivity as indicated by the parameter counts – ours (8.5 M) and 3DMolCSP (9.3 M). However, our pair representation is potentially more informative. It can directly capture interactions between the racemate and resolving agent, whereas 3DMolCSP would need to infer these interactions indirectly.

Notably, all Random Forest (RF) baseline models demonstrated similar performance, regardless of the molecular encoding employed. This held true whether using random numbers, Morgan fingerprints, or MAPC [16] – a fingerprint specifically developed to capture chiral features. This suggests that RFs may be less suited for this specific task, as their performance lags behind neural network-based approaches, even when provided with a chirally-informed representation. Crucially, RFs are not directly compatible with the two-step training scheme that provides a significant benefit to our neural network approach.

	Positive Class Metrics			Negative Class Metrics			Overall Metrics		
**Method**	**F1**	**Precision**	**Recall**	**F1**	**Precision**	**Recall**	**Bal. Acc.**	**AUC ROC**	**p-thresh.**
Random Forest Baselines									
Random Encoding	.03± .01	.04± .02	.02± .01	.938± .001	.923± .001	.953± .003	.488± .004	.45± .01	.047± .007
Morgan Encoding	.08± .02	.10± .02	.06± .01	.941± .001	.926± .001	.956± .002	.508± .006	.45± .01	.046± .004
MAPC Encoding [16]	.05± .004	.07± .004	.04± .004	.939± .001	.924± .0002	.955± .002	.498± .001	.421± .01	.037± .004
Our Approach									
Direct Classification	.07± .02	.09± .02	.05± .01	.940± .001	.925± .001	.955± .001	.505± .007	.47± .09	.22± .05
Direct Regression	.17± .03	.23± .04	.14± .02	.947± .002	.932± .002	.962± .002	.55± .01	.51± .01	.016± .005
Finetune Classification from Classification	.07± .01	.09± .01	.054± .008	.940± .001	.925± .001	.955± .001	.505± .004	.45± .05	.35± .09
Finetune Classification from Regression	**.23± .05**	**.31± .06**	**.19± .04**	**.951± .003**	**.936± .003**	**.966± .003**	**.58± .02**	.61± .06	.4± .1
Finetune Regression from Classification	.05± .01	.07± .02	.04± .01	.939± .001	.924± .001	.955± .001	.498± .006	.45± .04	.09± .02
Finetune Regression from Regression	.20± .03	.26± .04	.16± .02	.948± .002	.933± .002	.964± .002	.56± .01	.52± .01	.043± .006
3DMolCSP									
Direct Regression	.14± .06	.19± .08	.11± .05	.945± .004	.930± .004	.960± .004	.54± .03	.58± .06	.06± .01
Direct Classification	.05± .01	.06± .01	.036± .008	.939± .001	.924± .001	.954± .001	.495± .004	.49± .04	.3± .2
Finetune Regression from Regression	.08± .07	.10± .09	.06± .05	.941± .004	.926± .004	.956± .004	.51± .03	**.67± .05**	.14± .04
Finetune Classification from Classification	.08± .01	.10± .02	.06± .01	.941± .001	.926± .001	.956± .001	.508± .006	.57± .04	.74± .06
Finetune Regression from Classification	.07± .04	.09± .05	.05± .03	.940± .003	.925± .003	.955± .003	.51± .02	.59± .05	.24± .05
Finetune Classification from Regression	.10± .01	.13± .02	.08± .01	.942± .001	.927± .001	.957± .001	.518± .006	.57± .05	.5± .1

Table S3: Performance Comparison of Different Methods. The standard deviation was estimated by training five seeds for each method. The mean performance was calculated by taking the mean prediction across the five seeds.

S6 Causes of experimental error

In resolutions with a low fraction of racemate crashing out as solid (low w_{solid}), determining the enantiomeric enrichment directly from the solid is impractical. Instead, the enrichment is inferred from the mother liquor. If the enrichment in the liquor is $e.e._{\text{liquor}}$, the enrichment in the solid $e.e.$solid is:

$$e.e._{\text{solid}} = e.e._{\text{liquor}} \left(\frac{1}{w_{\text{solid}}} - 1 \right).$$

Any error in measuring $e.e._{\text{liquor}}$ gets amplified by $(1/w_{\text{solid}} - 1)$ when inferring $e.e.$solid. Below 20% w_{solid}, this amplification leads to more than 5 times higher experimental errors in $e.e._{\text{solid}}$ compared to $e.e._{\text{liquor}}$.

At higher w_{solid}, enrichment can be measured directly from the solid, avoiding this error amplification.

S7 Experimental details

S7.1 X-Ray structure deposition numbers

The structures shown in Figure 5 have the following CCDC deposition numbers:

- $\mathbf{C_1}$: 2408113
- $\mathbf{C_2}$: 2423476
- $\mathbf{C_3}$: 2408114
- $\mathbf{C_4}$: 2408115

For the prospective experiment, when multiple conditions led to crystallisation, X-Ray images were obtained only for the salts with the best figure of merit (highest mass fraction and enantiomeric excess). These structures and their deposition numbers are shown in the SFC-MS plots below. For convenience, we also include them here:

- **(S)-3-Phenylmorpholine** 2408118
- **(S)-spiro[2.3]hexane-1-carboxylic acid** 2408116
- **(S)-4,4,4-trifluoro-3-hydroxybutanoic acid** 2408117

S7.2 Prospective compound characterisation

Individual characterisation procedures are given in Table S4. The subsections that follow display individual chromatograms, and SFC-MS traces where a diastereomeric salt formed. For hits where X-ray crystallography was performed, SFC-MS traces of the solid are given. For the additional hits without X-ray crystallography, SFC-MS traces of the reaction liquors (solution phase) are given.

Image	Column	Mobile Phase A	Mobile Phase B	Gradient	Flow rate (mL/min)	Temp (°C)	Back Pressure (bar)	Detection
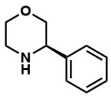	Phenomenex Lux Cellulose-4, 3 μm, 3.0x150 mm	CO_2	20 mM NH_3 in MeOH	Linear over 2.0 min from 10% B/A to 40% B/A	2.5	30	160	210-500 nm and SIM with APCI(+) ionization
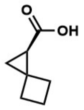	Daicel Chiralpak QN-AX, 5 μm, 4.6x150 mm	CO_2	20 mM NH_3 in MeOH	Isocratic at 20% B/A for 1.0 min, then linear over 1.5 min from 20% B/A to 30% B/A	2.5	40	160	210-500 nm and SIM with APCI(-) ionisation
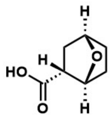	Daicel Chiralpak IK-3, 3 μm, 4.6x50 mm	CO_2	IPA	Isocratic at 5% B/A for 0.9 minutes followed by a linear gradient over 1.1 minutes from 5% B/A to 25% B/A	2.5	30	160	210-500 nm and SIM with APCI(-) ionization
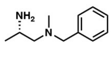	Daicel Chiralpak IC-3, 3 μm, 4.6x150 mm	CO_2	20 mM ammonium formate in MeOH	Isocratic at 25% B/A for 1.2 min, then linear over 2.0 min from 25% B/A to 50% B/A	3.5	25	160	210-500 nm and SIM with APCI(+) ionization
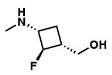	Daicel Chiralpak AD-3, 3 μm, 4.6x100 mm	CO_2	20 mM ammonium formate in MeOH	Isocratic at 8% B/A for 2.0 min	2.5	35	160	210-500 nm and SIM with APCI(+) ionization
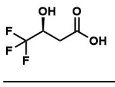	Phenomenex Lux Cellulose-4, 5 μm, 4.6x250 mm	CO_2	20 mM NH_3 in MeOH	Isocratic at 15% B/A for 3.5 min	3.5	35	160	210-500 nm and SIM with APCI(-) ionisation

Table S4: Characterisation procedure for the racemates used in the prospective experiment.

S7.2.1 (S)-3-phenylmorpholine

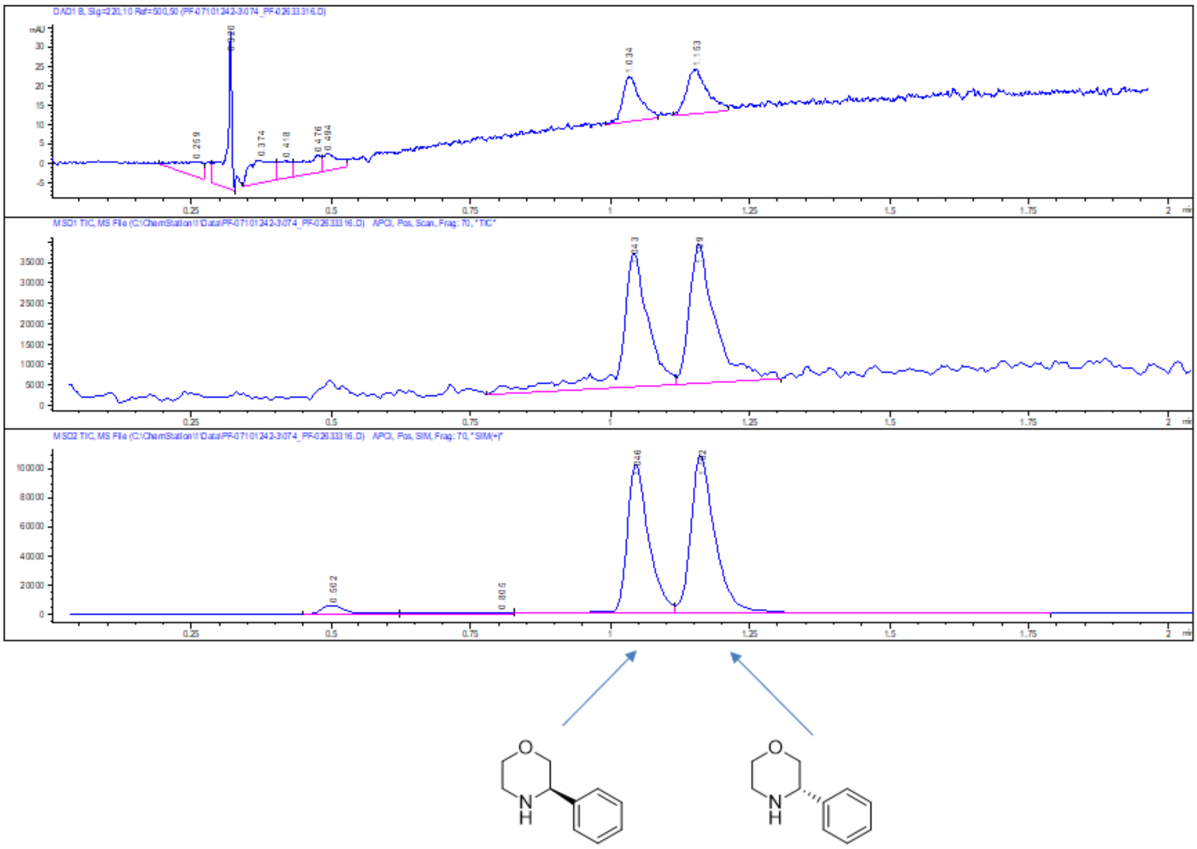

(*R*)-3-phenylmorpholine
retention time 1.04 min

(*S*)-3-phenylmorpholine
retention time 1.16 min

Figure S7: (S)-3-phenylmorpholine: Calibration chromatogram.

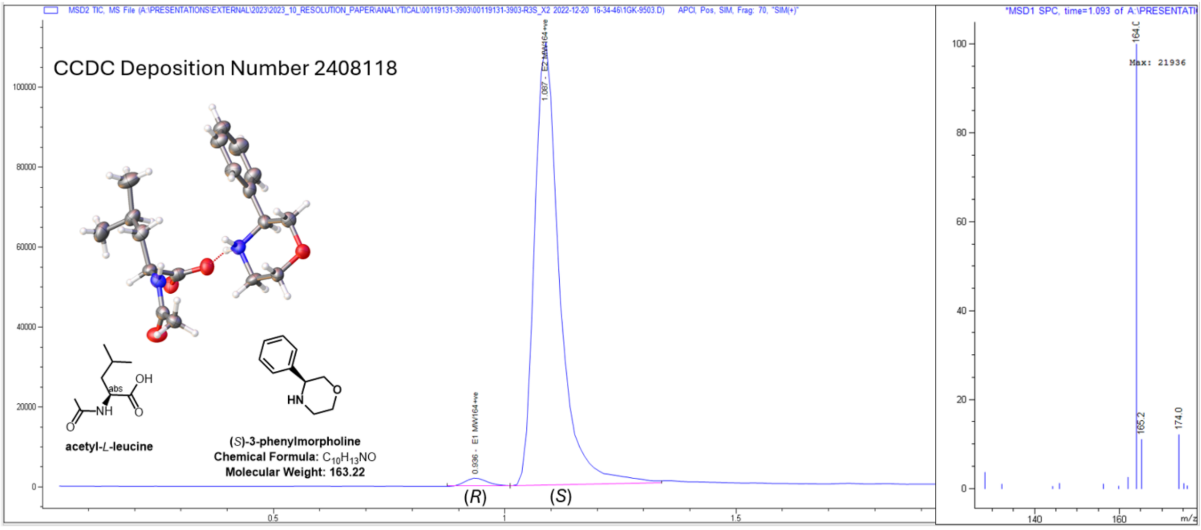

Figure S8: (S)-3-phenylmorpholine: Crystal SFC-MS trace.

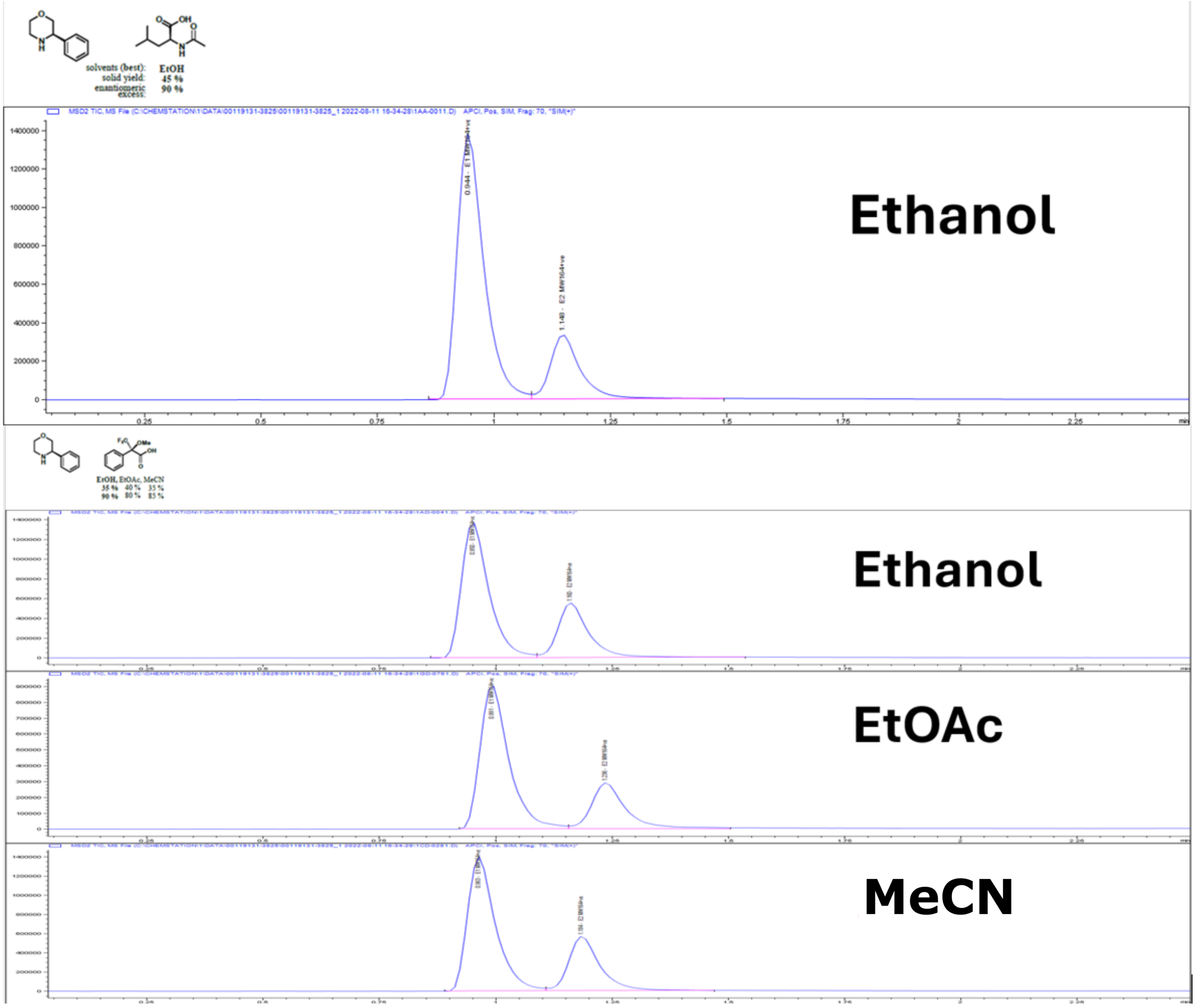

Figure S9: (S)-3-phenylmorpholine: Liquor SFC trace.

S7.2.2 (S)-spiro[2.3]hexane-1-carboxylic acid

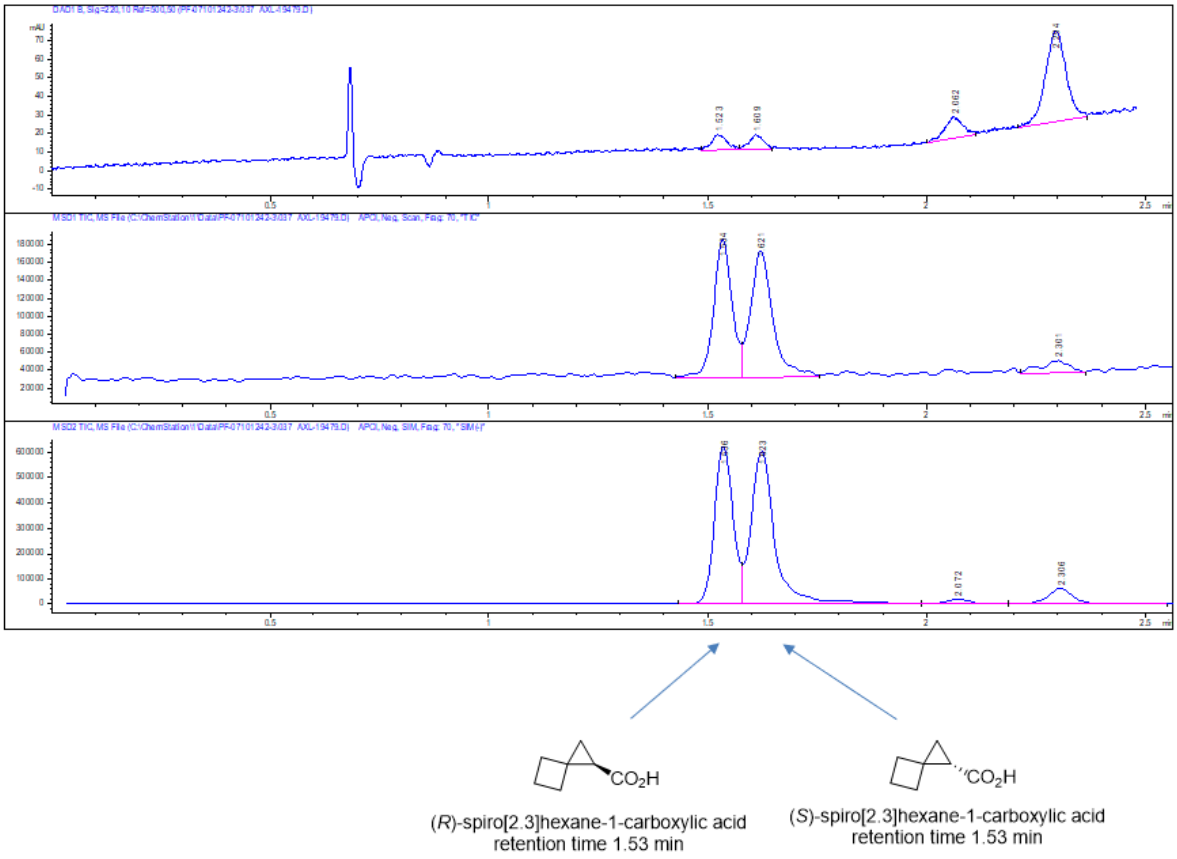

Figure S10: ((S)-spiro[2.3]hexane-1-carboxylic acid: Calibration chromatogram.

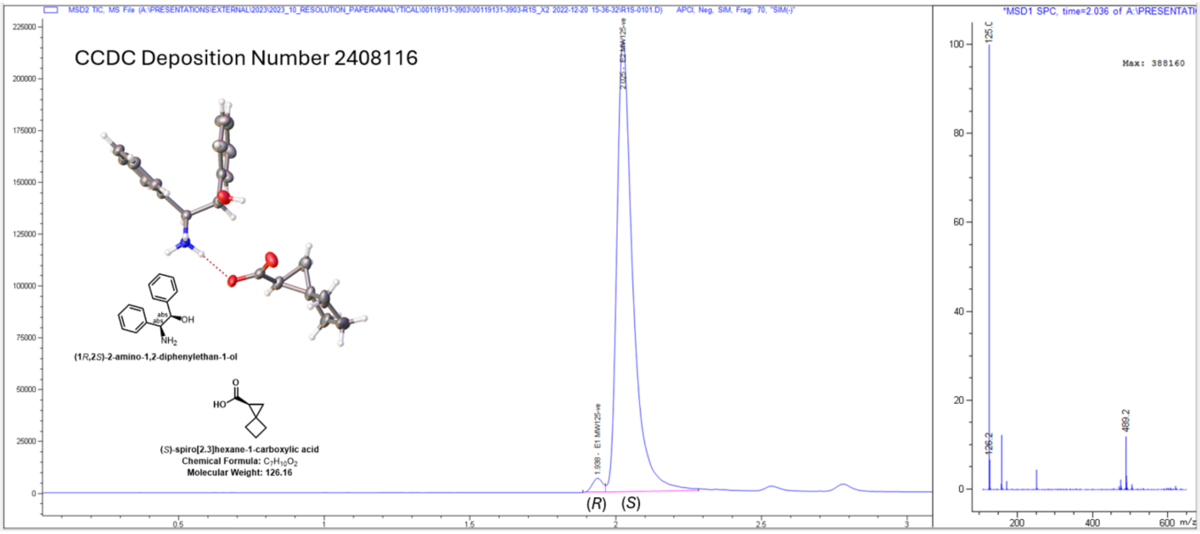

Figure S11: (S)-spiro[2.3]hexane-1-carboxylic acid: Crystal SFC-MS trace.

S7.2.3 *rel*-(1R,2R,4S)-7-oxabicyclo[2.2.1]heptane-2-carboxylic acid

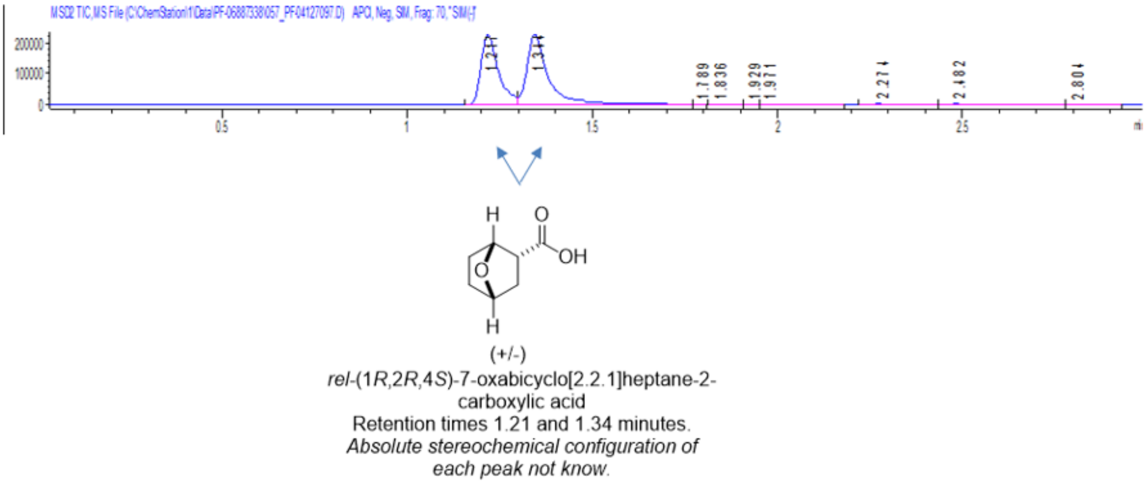

Figure S12: (*rel*-(1R,2R,4S)-7-oxabicyclo[2.2.1]heptane-2-carboxylic acid: Calibration chromatogram.

No diastereomeric salts generated.

S7.2.4 N1-benzyl-N1-methylpropane-1,2-diamine

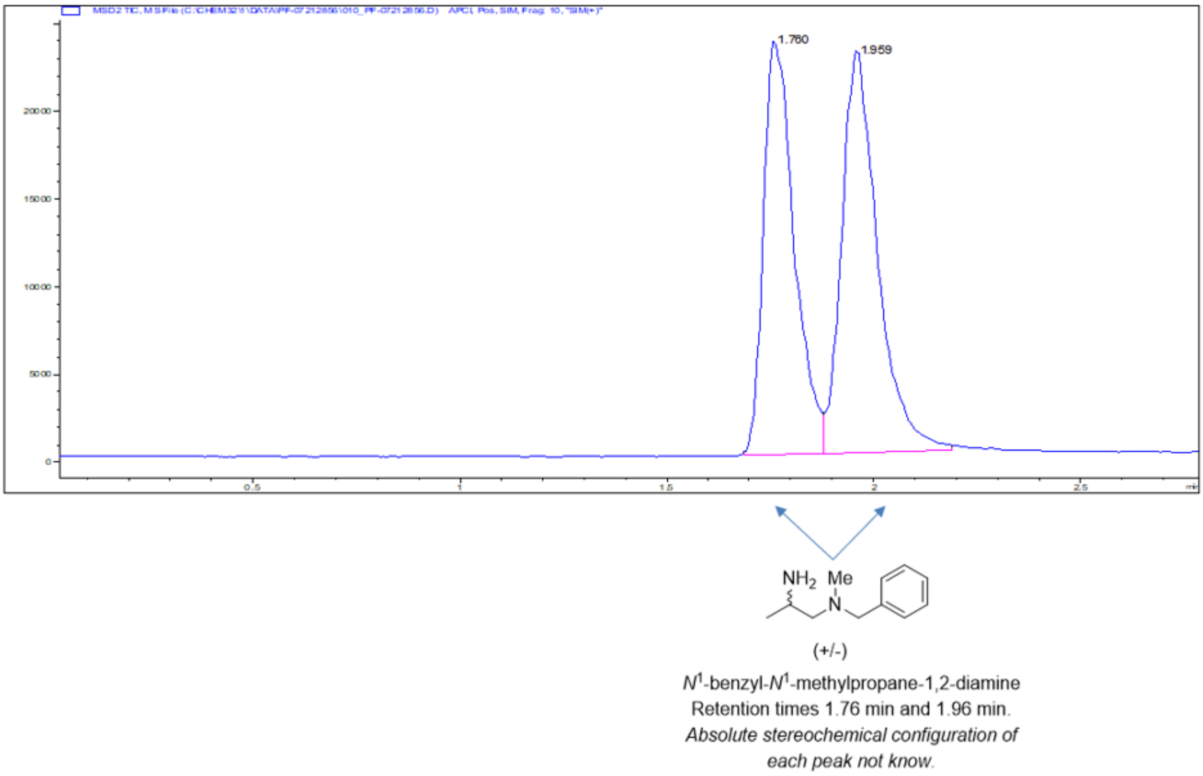

Figure S13: (N1-benzyl-N1-methylpropane-1,2-diamine: Calibration chromatogram.

No diastereomeric salts generated.

S7.2.5 ((*rel*-1S,2S,3S)-2-fluoro-3-(methylamino)cyclobutyl)methanol

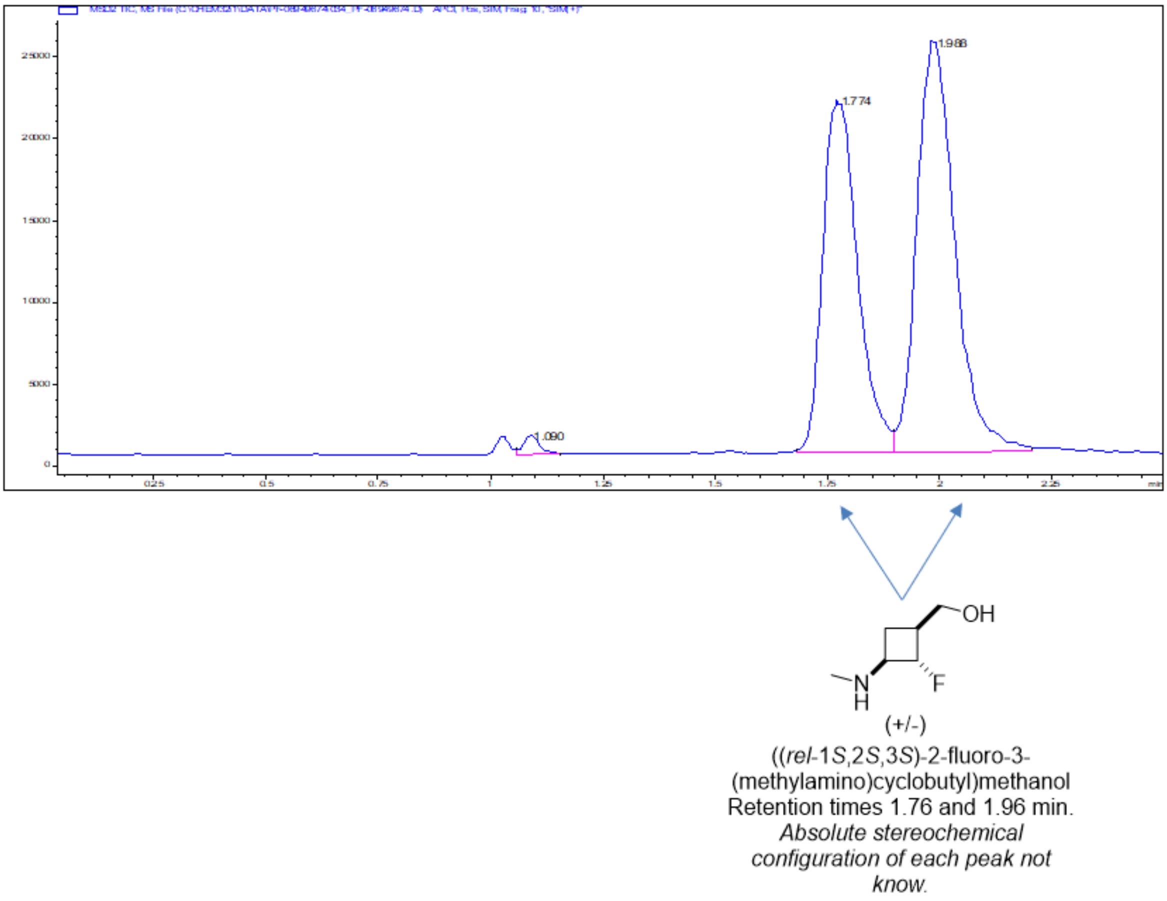

Figure S14: (*rel*-1S,2S,3S)-2-fluoro-3-(methylamino)cyclobutyl)methanol: Calibration chromatogram.

No diastereomeric salts generated.

S7.2.6 (S)-4,4,4-trifluoro-3-hydroxybutanoic acid

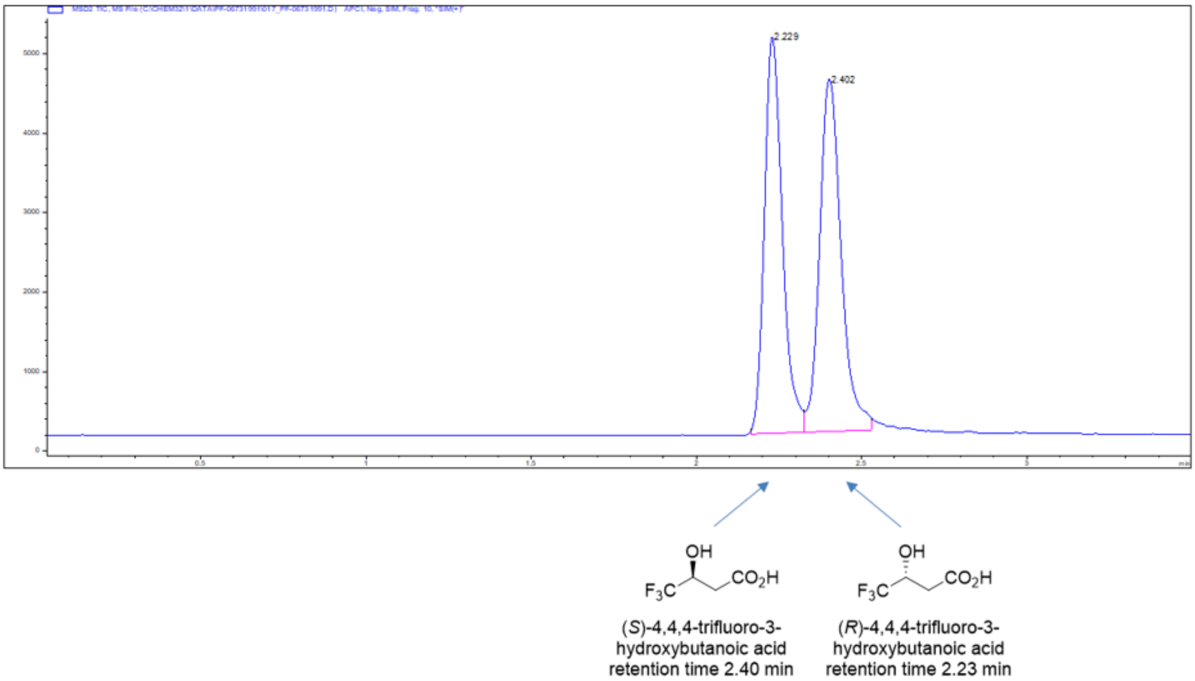

Figure S15: (S)-4,4,4-trifluoro-3-hydroxybutanoic acid: Calibration chromatogram.

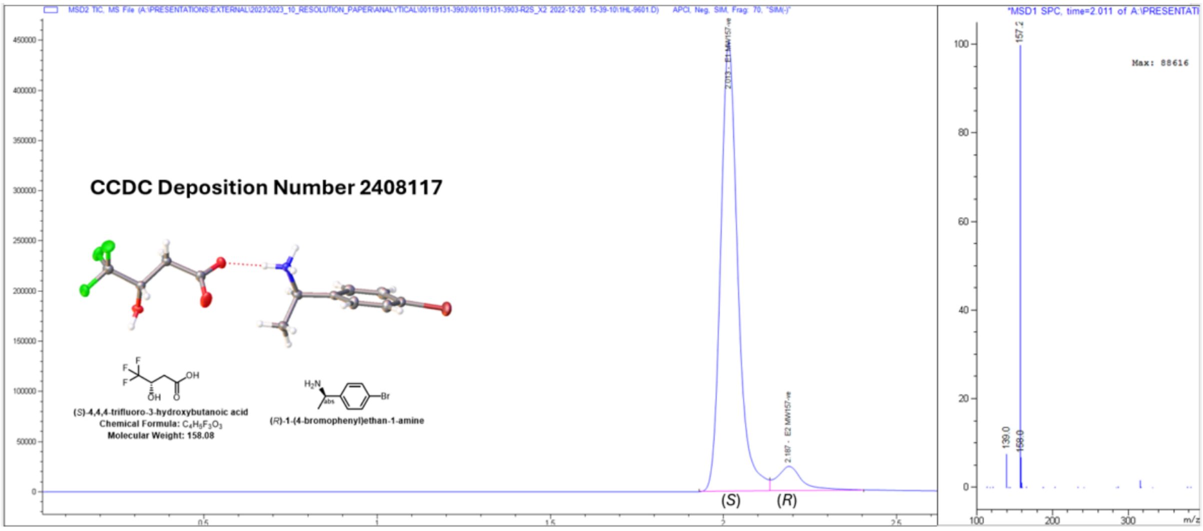

Figure S16: (S)-4,4,4-trifluoro-3-hydroxybutanoic acid: Crystal SFC-MS trace.

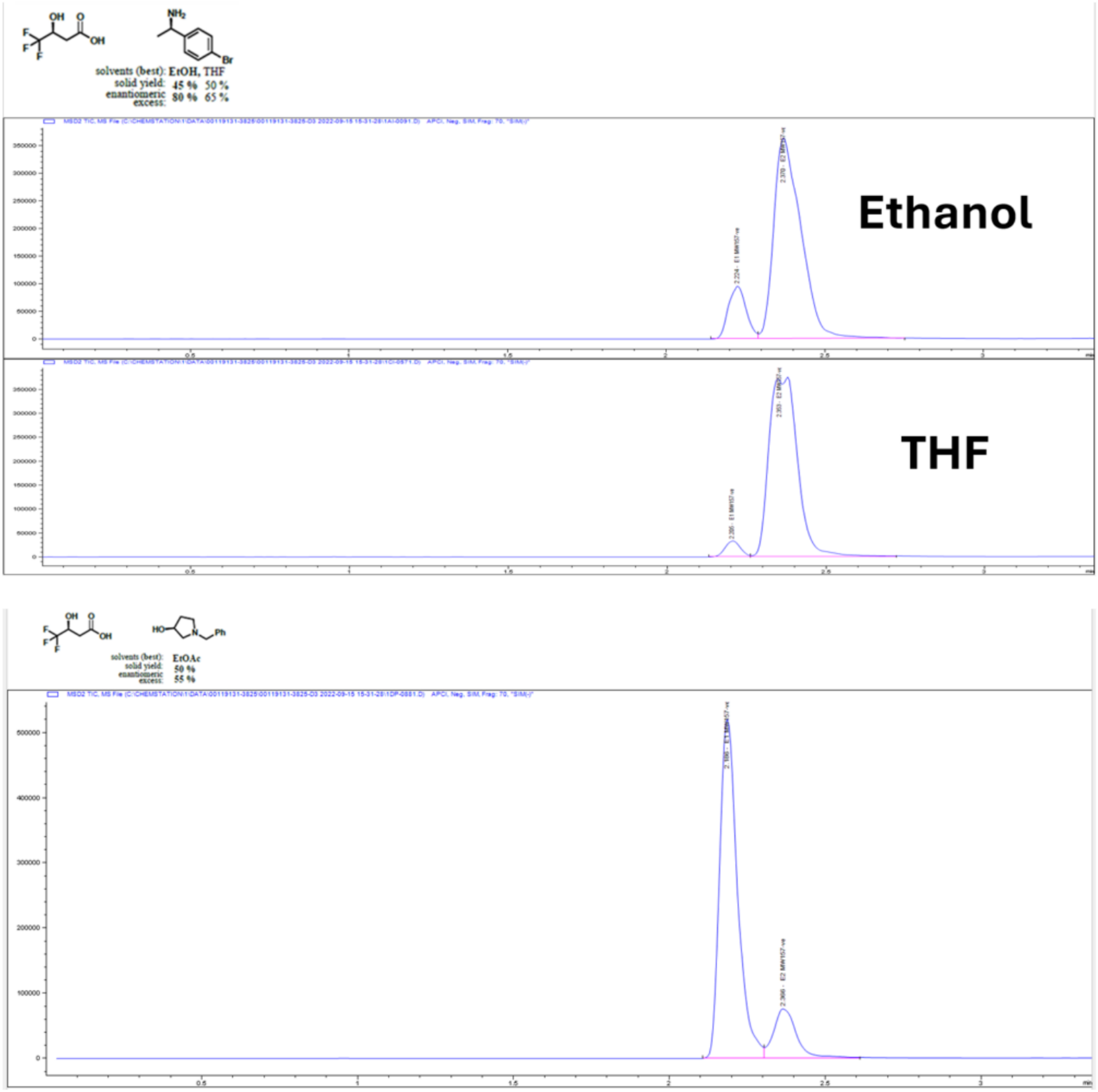

Figure S17: (S)-4,4,4-trifluoro-3-hydroxybutanoic acid: Liquor SFC trace.

Supplementary References

[1] COSMO*logic* GmbH & Co. KG. *COSMOtherm, Release 19.* http://cosmologic.de.

[2] H. Bourlard and Y. Kamp. "Auto-Association by Multilayer Perceptrons and Singular Value Decomposition". In: *Biological Cybernetics* 59.4-5 (1988), pp. 291–294. ISSN: 0340-1200, 1432-0770. DOI: 10.1007/BF00332918.

[3] Ashish Vaswani et al. "Attention Is All You Need". en. In: *arXiv:1706.03762 [cs]* (2017). arXiv: 1706.03762 [cs].

[4] Leslie N. Smith and Nicholay Topin. *Super-Convergence: Very Fast Training of Neural Networks Using Large Learning Rates.* 2018. DOI: 10.48550/arXiv.1708.07120. arXiv: 1708.07120 [cs, stat]. (Visited on 11/01/2023).

[5] Takuya Akiba et al. "Optuna: A Next-generation Hyperparameter Optimization Framework". In: *Proceedings of the 25th ACM SIGKDD International Conference on Knowledge Discovery and Data Mining.* 2019.

[6] F. Pedregosa et al. "Scikit-learn: Machine Learning in Python". In: *Journal of Machine Learning Research* 12 (2011), pp. 2825–2830.

[7] Jesse Davis and Mark Goadrich. "The Relationship between Precision-Recall and ROC Curves". In: *Proceedings of the 23rd International Conference on Machine Learning.* ICML '06. New York, NY, USA: Association for Computing Machinery, 2006, pp. 233–240. ISBN: 978-1-59593-383-6. DOI: 10.1145/1143844.1143874. (Visited on 03/05/2024).

[8] Takaya Saito and Marc Rehmsmeier. "The Precision-Recall Plot Is More Informative than the ROC Plot When Evaluating Binary Classifiers on Imbalanced Datasets". In: *PLOS ONE* 10.3 (2015), e0118432. ISSN: 1932-6203. DOI: 10.1371/journal.pone.0118432. (Visited on 03/05/2024).

[9] K. R. Müller et al. "A Numerical Study on Learning Curves in Stochastic Multilayer Feedforward Networks". In: *Neural Computation* 8.5 (1996), pp. 1085–1106. ISSN: 0899-7667. DOI: 10.1162/neco.1996.8.5.1085. (Visited on 03/06/2024).

[10] Christopher A Lipinski et al. "Experimental and Computational Approaches to Estimate Solubility and Permeability in Drug Discovery and Development Settings1". In: *Advanced Drug Delivery Reviews.* Special Issue Dedicated to Dr. Eric Tomlinson, Advanced Drug Delivery Reviews, A Selection of the Most Highly Cited Articles, 1991-1998 46.1 (2001), pp. 3–26. ISSN: 0169-409X. DOI: 10.1016/S0169-409X(00)00129-0. (Visited on 01/21/2025).

[11] Simon Axelrod and Rafael Gómez-Bombarelli. "GEOM, Energy-Annotated Molecular Conformations for Property Prediction and Molecular Generation". In: *Scientific Data* 9.1 (2022), p. 185. ISSN: 2052-4463. DOI: 10.1038/s41597-022-01288-4. (Visited on 01/21/2025).

[12] Anna Gaulton et al. "ChEMBL: A Large-Scale Bioactivity Database for Drug Discovery". In: *Nucleic Acids Research* 40.Database issue (2012), pp. D1100–1107. ISSN: 1362-4962. DOI: 10.1093/nar/gkr777.

[13] G. Richard Bickerton et al. "Quantifying the Chemical Beauty of Drugs". In: *Nature Chemistry* 4.2 (2012), pp. 90–98. ISSN: 1755-4349. DOI: 10.1038/nchem.1243. (Visited on 01/22/2025).

[14] Yuhui Hong et al. "Enhanced Structure-Based Prediction of Chiral Stationary Phases for Chromatographic Enantioseparation from 3D Molecular Conformations". In: *Analytical Chemistry* 96.6 (2024), pp. 2351–2359. ISSN: 0003-2700. DOI: 10.1021/acs.analchem.3c04028. (Visited on 01/31/2025).

[15] *RDKit: Open-source cheminformatics.* http://www.rdkit.org. [Online; accessed 27-June-2021].

[16] Markus Orsi and Jean-Louis Reymond. "One chiral fingerprint to find them all". In: *Journal of Cheminformatics* 16.1 (May 2024), p. 53. ISSN: 1758-2946. DOI: 10.1186/s13321-024-00849-6. URL: https://doi.org/10.1186/s13321-024-00849-6 (visited on 05/27/2025).
